# Children’s Health and Typology of Family Integration and Regulation: A Functionalist Analysis

**DOI:** 10.3390/children10030494

**Published:** 2023-03-02

**Authors:** Xiaozhao Yang, Chao Zhang

**Affiliations:** School of Journalism and Communication, Sun Yat-sen University, Guangzhou 510275, China

**Keywords:** self-reported health, child–parent relationship, functionalism, family regulation

## Abstract

Rationale: Children’s health is conventionally studied as an ultimate consequence resulting from various social and biological processes that jointly channel the risk factors and pathogens toward an individual health outcome. What is currently neglected is the rich tradition of a functionalist analysis of children’s health as a necessary function in the family institution. Children’s health may be associated with how children are integrated into the family’s core functioning and how parents regulate children’s behaviors. Methods: The current study used a cross-sectional sample of 891 parents from 2018 southern Jiangsu and surveyed information about children’s health and family activities. Employing a latent class analysis, we established four types of families based on children’s integration and parental regulation: loose, free, pressed, and concerted. Results: The regression results showed that a child’s health is associated with the concerted family type (OR = 3.6, *p* < 0.05), indicating the necessary functionality of health in heavily regulated and mobilized families. Conclusion: This study broadens the perspective on children’s health by ushering back functionalism and placing health in its social implications.

## 1. Introduction

### 1.1. Children in the Family Institution

The boundary between childhood and adulthood has become increasingly ambiguous and the social roles assigned to each are increasingly opaque [1]. If anything, the conventional relationship between adults and their children has melted in the air as a myriad of formative socio-demographic conditions for traditional adulthood have permanently changed, including the delayed entrance into the labor force, delayed or entirely extinct marital union, earlier romantic relationship, digitized social interaction, and the rise of a precarious gig economy, among others.

Functionalism is a paradigm that views each of the components in an organic institution, such as family or religion, operating latently or explicitly to sustain the survival of the institution. Functionalism posits that all constituents of a society—institutions, roles, norms, etc.—serve their purpose for the long-term survival of the society [2]. From a functionalist perspective, the family institution survives on the basis of its own functioning components, including the health of family members. Instead of a “product” of the family, the functionalist perspective argues that children are necessary components contributing to the functioning and survival of the family institution by reproducing the existing relations and resources in the family [3,4,5]. Children’s health, rather than as an outcome, is regarded as an integral component for facilitating the function of the family. A child is integrated into the family by participating as an agent in family activities, including leisure activities, emotional correspondence, and labor activities [6,7].

Recently, the children’s agency in the family institution has received rising attention in academia [4,8,9,10,11]. Some scholars have classified parental style into four general types: neglectful, permissive, authoritarian, and authoritative [12,13,14,15]. Certain parenting styles contribute to different child development outcomes, such as delinquency, academic performance, and mental health [16,17,18,19].

### 1.2. Children’s Health and the Family Institution

A child who may not perform the normative social role due to irresistible non-personal causes unwillingly disrupts the integration between him/her and other members, and undermines the function of his/her family in society [20]. The most common among these types of irresistible causes is disease and disability [21,22,23]. Talcott Parsons was the first to formulate the patient’s role as an integrated and necessary part of a functional society [24]. Scholars later introduced the intersectional perspective based on identities—racial, gender, ageism—to studying how health shapes our normative role in an institution such as family [25,26,27,28].

In the world of the family institution, unhealthy children may not be expected to partake in certain social activities and may be exempted from family obligations that the family would otherwise prescribe [29]. Most contemporary studies on social activities, manifested in various forms pertaining to their respective conceptional traditions, such as social capital theory, social support, and routine activity theory, seek to establish a directional causal relationship between health and social integration [30,31,32]. Children with health problems may be discouraged from participating in family decision and collective activities by their caregivers [33]. People suffering from disease or general infirmity are expected by the social norm to proactively seek to improve their current infirmary condition and resume their previous social functions. When they fail to achieve this, sanctions and stigma befall their existing functions in the social relation nexus and they tend to be excluded from further participating in other social activities [22,24].

### 1.3. Gaps in the Literature

**Figure 1:**
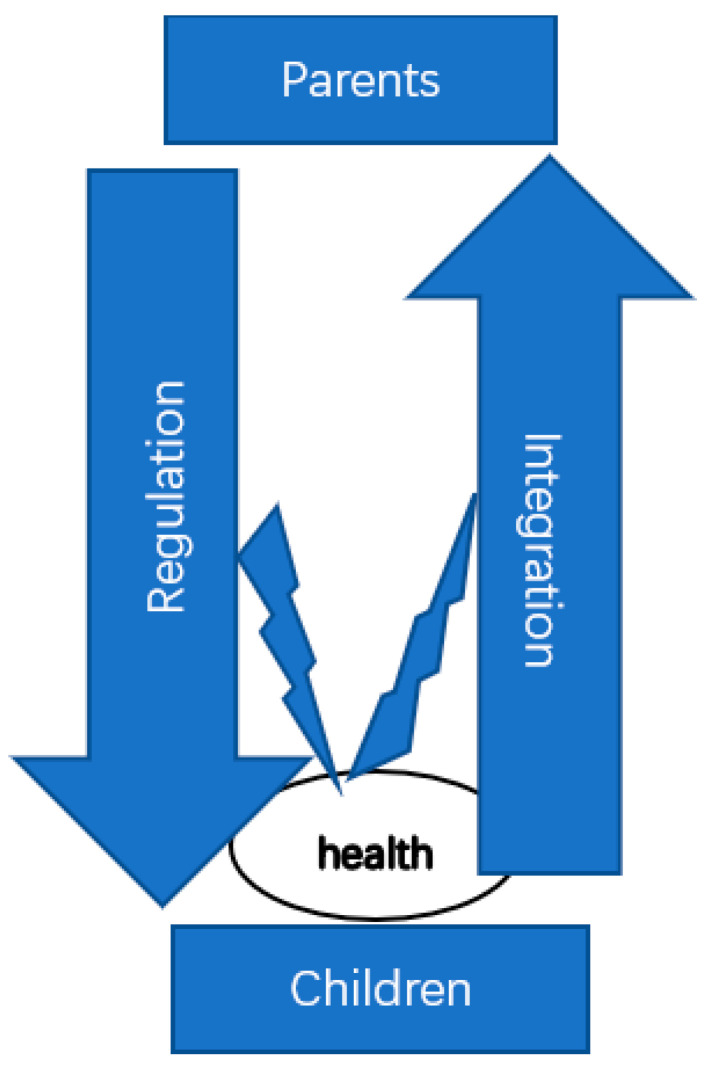
Conceptual Diagram for the Functions of Children’s Health in Family Relations.

While a considerable portion of the literature on child and health has noticed the importance of the family environment, very few studies have considered child health as a condition for social integration in the family. The current study builds on a sample of 891 parents of young children and investigates how social integration is an embedded function of health. Specifically, we proposed to examine the focal research question on the functional role of health in children’s familial and extra-family social integration: does health provide function for family solidarity in terms of child–parent integration and parent–child regulation; is health functional for participating in social activities outside of a child’s family? We illustrate this framework with Figure 1. Here, parents and children mutually regulate and integrate into each other, while the health of the children is associated with the extent to which such regulation and integration can fully realize.

## 2. Methodology

### 2.1. Sample

The individual-level family data of this study come from a field survey from December 2018 to January 2019. The empirical survey was carried out among the parents of senior primary students from four urban ordinary primary schools in two coastal cities in eastern China. Based on the population of the city and assuming simple random sampling, the desired minimum sample size of was determined according to the formula: $n=\frac{1.96^{2}*0.5*0.5}{1+1.96^{2}*0.25}N=384$. The investigators reached to the administrators and teachers at four primary schools. The administrators in each class unit then distributed the survey invitation to students, who, in turn, would invite their parents to respond to a self-administered survey. Parents reserve the right to voluntarily participate or withdraw at any point of the survey. Eligibility: pupils who did not attend the school at the day or could not inform their parents of the survey did not contribute to the data collection. Although the survey did not initiate a pilot test, its questionnaires comprised standardized format for most questions. The measurement includes household size, parent–child relationship, family integration, family regulation, child’s social participation, child’s healthy and demographic variables, etc. Finally, 900 samples were collected, and the invalid samples were listwise removed. The total number of valid samples was 891, which constituted the basic database for this study. The equivalent to the Institutional Review Board that approved the study design was an ethics committee of Soochow University.

### 2.2. Measurement

Health status of the child was self-reported by parents to the question “your child’s health condition is: very bad, somewhat bad, okay, somewhat good, very good”. We concatenated categories from somewhat good to very good into healthy status, and the remaining three were combined into unhealthy status.

Family integration was measured by summating three questions. For questions “when your family decide the child’s extra-curriculum activities”, “when your family makes major financial decisions”, “when your family makes familial decisions”, each question yields 1 point if the parent chose “mainly decided by the child” or “will incorporate child’s decision”. The score of family integration ranges from 0 to 3.

Family regulation was measured by summated average of parental involvement in the child’s behaviors in the following categories: making friends at school, making friends outside school, dressing, using the Internet, watching TV. Regulation score also ranges from 0 to 3 after we divided the raw score into equidistant four categories.

Control variables included household size, gender, parent’s education, parent’s subjective class, parent’s spousal relation, the child’s willingness to participate in extra-curriculum activities, and the child’s actual participation in extra-curriculum activities.

### 2.3. Analytical Strategy

To categorize population into conceptually distinct typologies based on the two dimensions of family structure—integration and regulation—we used latent class analysis that a posteriori creates group labels based on the observed data. Because the group labels are created based on the best fit from maximum likelihood function, we selected the best model based on fit indices such as BIC and log-likelihood change [34]. The estimation of the probability of belonging to each class y expresses the joint probability of the K-class-specific probability, conditioning on a set of variables; that is:

$$P\left(y|x\right)=\overset{k}{\underset{k=1}{\prod }}P\left(y_{k}|x\right)\pi_{k}$$

With the probability of each latent class for individual calculated, we used the cut-off threshold of 0.5 to assign class membership. Logistic regression was then applied to children’s health status with the latent classes of family integration and family regulation as the key independent variables. Analyses were conducted with Stata 16.

## 3. Results

In Table 1, descriptive statistics of the sample composed of 891 respondents demonstrate the characteristics of key study variables and the demographic information. The sample is equally divided between male and female children, with 47.6% being females. The vast majority (90.8%) of the children are reported to be healthy or very healthy by their parents, leaving one tenth of the children in the categories below and including “okay”. The average household size in this study is 2.45, which is within the typical range of household size that was formed under the one-child policy. The average highest parent education is 4.7, corresponding approximately to the vocational school. The parents’ subjective class on average is unsurprisingly 3.09, corresponding to “at the average level”. The mean relational conflict between spouses is 1.49, which is between “very harmonious” and “relatively harmonious”. Out of four levels, children’s integration into the family averages at 1.49, and their level of being regulated by parents averages at 1.66. The average level of social participation among children is 2.29, and their willingness to participate averages at 2.13.

The children’s integration into family decisions, management, and arbitrations conceptually refers to a down-to-top pipeline of forming solidarity within the family domain, and the level of regulation imposed on children’s decisions and arbitrations represents a top-to-down channel of enforcing family solidarity from parents as the administrators of the organization. Each dimension of family solidarity as a functional representation of an organization is measured and quantified into four levels. The interactive juxtaposition of these two dimensions—integration and regulation—produces several idealtypical classes of intra-family relational types. In the left panel of Table 2, the entire sample can be divided into four groups along the levels of regulation and integration, with values smaller than and equal to 1 as the cut-off for low and high levels. Then, we arrive at the right panel of Table 2 as a conceptual construction of family solidarity types: the “loose” type has low levels in both regulation and integration; the “free” type has children highly integrated into the family but receiving little regulation; the “pressed” type is highly regulated and weakly integrated; the “concerted” type is high in both regulation and integration.

The grouping in Table 2 springs from conceptual construction. Now, we examine the empirical fitness and validity of such constructs with a latent class analysis. Using integration and regulation as manifest indicators regressing on a categorical latent construct, Table 3 shows that the four-group construction retains the highest fitness and parsimony. Such a four-group latent class outcome is described in Table 4. Class 1 is low in both regulation and integration, corresponding to the “loose” ideal type in Table 2. Class 2 is low in terms of regulation but high in integration, representing the “free” ideal type. Class 3 is high in regulation but low in integration, representing the “pressed” type, while class 4 is high in both regulation and integration.

**Figure 2:**
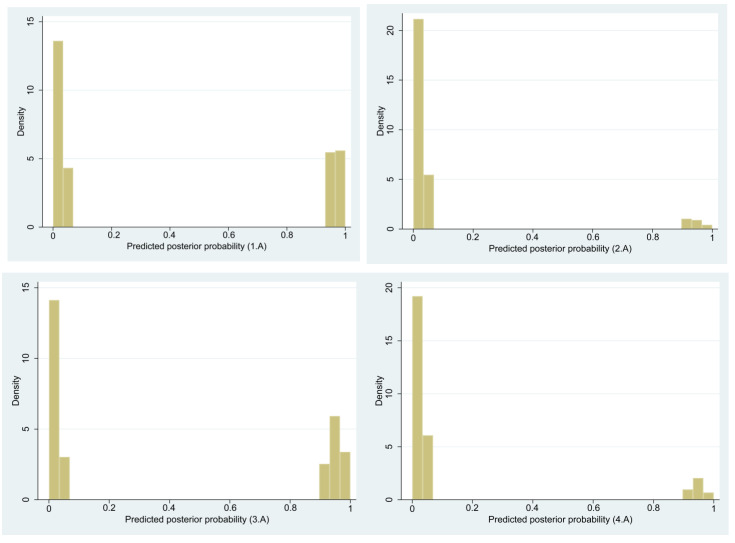
Bimodal distributions of the estimated probability of belonging to each latent class, from class 1 to class 4 (from 1.A to 4.A). N1 (*p* > 0.5) = 340, N2 (*p* > 0.5) = 73, N3 (*p* > 0.5) = 364, N4 (*p* > 0.5) = 114.

The probability of each individual belonging in each latent class indicates the certainty of assigning individuals to each construct. Figure 2 demonstrate that our latent classes are highly deterministic and the certainty of assignment group membership is almost absolute. The bimodal shapes of probability distributions indicate that each respondent is either a member of one latent class or not a member, with little ambiguity and overlap. When 0.5 is used as the cut-off of the posterior probabilities, the sizes of latent classes are exactly the same as that of the idealtypical classes in Table 2, confirming the empirical validity of the conceptual construction of family types.

Table 5 contains logistic models regressing on health by the step-wise inclusion of independent variables. Model 1 shows that child health is positively associated with more social participation (OR = 1.94, *p* < 0.01), where a healthy child is 1.94 times more likely to have one greater level of social participation compared to an unhealthy child. Compared to the “loose” family type, the concerted family is associated with child health by 3.6 times the odds ratio. Children in the concerted families are 3.6 times more likely to be healthy compared to children in the loose families. Including child gender and willingness to participate in model 2 does not change the precedent coefficients greatly. A female child, however, is more likely to be healthy (OR = 1.67, *p* < 0.05). Finally, several variables measuring family characteristics were introduced to model 3. Among them, the relational conflict among parents is negatively associated with health (OR = 0.62, *p* < 0.001). A one-unit increase in parental conflict is associated with a 38% lower chance of reporting a healthy child. Family characteristics accounted for the association between family type and child health, as its coefficients turned non-significant at an alpha level of 5%. The association between child health and social participation remains significant (OR = 1.84, *p* < 0.05).

## 4. Discussion

The current study employed a structural functionalist theoretical framework to understand the intricate role of children’s health in the functioning and integration of the family, as well as in children’s participation in social activities outside the family domain. This model builds upon a tradition in medical sociology that contrasts against the prevailing biomedical model of health as an independent agent in the causal chain from social determinants to individual health outcomes. Instead, medical sociology views health not only as a product of social determinants in the multilevel ecological layers full of risk factors but also emphasizes health as a function and organic part that enables the integration and solidarity of a societal unit, such as a family or a corporate [21]. People with a satisfactory level of health perform daily tasks defined by their social role; thus, their health is an essential component of a functional social role.

The literature on family and child development has touched base on how the style and intensity of integration and regulation within a family are associated with behavioral and social outcomes among children. Studies have elaborated the typology of parenting; for example, into authoritative, authoritarian, permissive, and uninvolved [11]. Authoritative parenting that combines child agency and certain levels of regulation may benefit the child in terms of deviant behaviors and academic outcomes. Recent studies on concerted parenting point to a similar finding that underlies our argument: the functional operation of a family organization in a competitive environment requires the active participation of multiple actors, including parents and children [18,35,36]. Therefore, in line with the Durkheimian tradition, we argue that both integrations of children in the family and regulation by parents towards children are necessary components in understanding the family as a functional unit.

Based on the functionalist perspective of family, this study argued that family could be conceptually classified into four types based on the levels of integration and regulation: the loose, the free, the pressed, and the concerted type. These conceptional ideal types are validated with empirical data from 891 parental reports of family relations in a 2018 Chinese sample. In the subsequent regression analyses, we found that reported health is positively associated with the concerted type of family compared to the loose type, with a very large odds ratio of 3.6. Only the coefficient was reduced to being non-significant after controlling for family characteristics. Social participation is significantly associated with the reported health of children throughout all models. These findings indicate that children’s health is an integral component for the functional operation of the family in both domestic and extra-family activities. A child with above average health lives in a concerted family where the child’s agency is encouraged and actively incorporated into family decisions; meanwhile, parents’ regulation on the child is also maintained at a considerable level. The same child with good health also participates in more social activities, and more intensively so.

The overall level of child health tends to be stable in a society, but children with a bad health condition make their families sacrifice more resources and energy in maintaining their wellbeing. Furthermore, the family will suffer more than physical and material loss as it reorients its organizational structure and change the types of regulation and integration among family members. In the Chinese context, predominant single-child households with a sick child face serious challenges regarding their own integration into the broader society. In short, families with a sick child may not perform their full function and risk the cohesion between different social components.

One must bear in mind that this study employs a functional theory in regard to the relationship between children’s health and their social integration, instead of a causal mechanism. The functional analysis is concerned with the constitution of a family organization by certain integral components each contributing a function to the operation of the family; thus, it is not preoccupied with the direction of an effect. Each functional component of the family, including the children’s health, family finance, and spousal relation, is topologically connected to other components so that they synergistically form the functions of a family. Children’s health may lead to a more concerted family type and more active social participation, but it may well be the reverse causality that a concerted family improves its children’s health outcome. However, this study has no intention to engage in the mechanism discussion on what causes or is caused by children’s health. Overall, this study adds to the child development literature by revealing that children’s health is functionally necessary for a family with higher levels of integration and regulation and for closer social engagement through social participation.

## 5. Limitations

The current study was subjected to several limits that restrained the generalizability of the results to all contexts. First, this study employed the functionalist perspective that regards health wellbeing as a functional element for the maintenance and performance of the family unit. Scholars from a different theoretical tradition may see health only as an outcome caused by the structure and wellbeing of the family institution. Second, this study was based on a cross-sectional survey, and does not intend to inform readers of the causality between children’s health and family structure. Third, the sample came from a specific cultural region of China and cannot extrapolate the results to the heterogenous country.

## 6. Conclusions

Adopting a functionalist analytic framework, the current study investigates children’s health as a necessary component for the functioning of the family institution and argues that children’s health is closely integrated in how parents interact with the children. Using a 2018 sample from the southern Jiangsu province of China, we employed a latent class analysis to categorize four types of a parent–child relationship: the loose, free, pressed, and concerted types. Multivariate logistic regressions showed that the concerted type, in which parents moderately regulate the behavior of the children, is associated with a better level of children’s health. The results suggest that children with good health render it possible for a family to develop a concerted parent–child relationship and function as an integrated unit. Such a finding calls for policymakers to consider that children’s health has consequences beyond medical and healthcare relevancy. Children’s health may further matter for a functioning family institution and, ultimately, a well-balanced and integrated society in which individuals find solidarity and cohesion among themselves.

## Figures and Tables

**Table 1:** Sample descriptive statistics.

	Mean (s.d.)	N (%)
Gender (female)		424 (47.6%)
Healthy		809 (90.8%)
Parental status		
-father		178(20%)
-mother		713(80%)
Household size	2.45 (0.91)	
Parent education	4.7 (1.43)	
Parent subjective class	3.09 (0.59)	
Parent relation	1.49 (0.71)	
Family integration	0.85 (0.83)	
Family regulation	1.66 (1.14)	
Child’s social participation	2.29 (0.55)	
Child’s participation willingness	2.13 (0.57)	

**Table 2:** Number of people by levels of regulation and integration in the family sphere.

	Regulation→				
Integration→		0	1	2	3
	0	75	97	73	104
	1	76	92	78	109
	2	28	32	30	63
	3	6	7	3	18
		low		High	
	low	“Loose” 340		“Pressed” 364	
	high	“Free” 73		“Concerted” 114	

**Table 3:** Model fit evaluation information.

	Log-Likelihood	BIC
1 group	−2483	4994
2 groups	−2339	4726
3 groups	−2327	4722
4 groups	−2282	4652
5 groups	−2271	4653

**Table 4:** Four-class model by latent class analysis.

N = 891	Class 1 (Loose)	Class 2 (Free)	Class 3 (Pressed)	Class 4 (Concerted)
Intercept	1	−1.49 (0.15)	0.04 (0.08)	−1.06 (0.12)
Regulation	0.58 (0.03)	0.55 (0.07)	2.58 (0.03)	2.72 (0.05)
Integration	0.50 (0.03)	2.08 (0.08)	0.52 (0.03)	2.10 (0.06)
Probability	0.38 (0.02)	0.09 (0.11)	0.40 (0.02)	0.13 (0.01)

**Table 5:** Logistic regression results in odds ratios with standard errors and 95% confidence intervals.

Criterion Variable = Healthy	Model 1 (n = 891)		Model 2 (n = 891)		Model 3 (n = 891)	
Family type (ref = loose)						
-free	0.96 (0.40)	0.43–2.16	0.97 (0.41)	0.43–2.20	0.82 (0.35)	0.36–1.88
-pressed	1.27 (0.33)	0.76–2.13	1.28 (0.34)	0.76–2.14	1.16 (0.31)	0.69–1.96
-concerted	3.60 (2.23) *	1.07–12.2	3.60 (2.24) *	1.06–12.18	2.97 (1.86)	0.87–10.2
Social participation	1.94 (.49) **	1.18–3.20	1.93 (0.50) *	1.16–2.30	1.84 (0.50) *	1.08–3.12
Participation willing			0.93 (0.21)	0.60–1.44	0.97 (0.22)	0.62–1.51
Child sex (ref = male)			1.67 (0.41) *	1.03–2.69	1.69 (0.41) *	1.04–2.73
Parent subjective class					1.21 (0.24)	0.82–1.79
Parent education					0.92 (0.08)	0.77–1.10
Household size					0.97 (0.13)	0.74–1.27
Parent relation					0.62 (0.09) ***	0.46–0.83
Pseudo R2	0.03		0.04		0.07	
Correctly classified †	90.8%		90.8%		90.6%	

† Pr(D|+) + Pr(~D|−), Pr > 50%. * *p* < 0.05, ** *p* < 0.01, *** *p* < 0.001.

## Data Availability

Data sharing not applicable.

## References

[1] Song C., Zhang C. (2021). The Public’s Supportive Attitude towards the Social Inclusion of Children with Special Needs: Theory and Experience. China Nonprofit Rev..

[2] Durkheim E. (1893). De la Division du Travail Social: Étude sur L’organisation des Sociétés Supérieures.

[3] Cherlin A.J. (2014). Labor’s Love Lost: The Rise and Fall of the Working-Class Family in America.

[4] Maccoby E.E. (1994). The Role of Parents in the Socialization of Children: An Historical Overview.

[5] Grusec J.E., Lytton H. (1988). Socialization and the family. Social Development.

[6] Hay C., Forrest W. (2006). The Development Of Self-Control: Examining Self-Control Theory’s Stability Thesis*. Criminology.

[7] Kalmijn M., De Graaf P.M. (2012). Life Course Changes of Children and Well-being of Parents. J. Marriage Fam..

[8] Zhang C. (2017). ‘Nothing about us without us’: The emerging disability movement and advocacy in China. Disabil. Soc..

[9] Silverstein M., Gans D., Lowenstein A., Giarrusso R., Bengtson V.L. (2010). Older parent–child relationships in six developed nations: Comparisons at the intersection of affection and conflict. J. Marriage Fam..

[10] Sawyer S.M., Azzopardi P.S., Wickremarathne D., Patton G.C. (2018). The age of adolescence. Lancet Child Adolesc. Health.

[11] Dhingra P. (2020). Hyper Education: Why Good Schools, Good Grades, and Good Behavior Are Not Enough.

[12] Bengtson V., Giarrusso R., Mabry J.B., Silverstein M. (2002). Solidarity, Conflict, and Ambivalence: Complementary or Competing Perspectives on Intergenerational Relationships?. J. Marriage Fam..

[13] Silverstein M., Bengtson V.L., Lawton L. (1997). Intergenerational solidarity and the structure of adult child-parent relationships in American families. Am. J. Sociol..

[14] Kirkpatrick L.A., Davis K.E. (1994). Attachment style, gender, and relationship stability: A longitudinal analysis. J. Pers. Soc. Psychol..

[15] Shaver P.R., Brennan K.A. (1992). Attachment Styles and the “Big Five” Personality Traits: Their Connections with Each Other and with Romantic Relationship Outcomes. Personal. Soc. Psychol. Bull..

[16] Hirschi T. (1977). Causes and Prevention of Juvenile Delinquency. Sociol. Inq..

[17] Morton P.M., Ferraro K.F. (2020). Early Social Origins of Biological Risks for Men and Women in Later Life. J. Health Soc. Behav..

[18] Schroeder R.D., Higgins G.E., Mowen T.J. (2014). Maternal Attachment Trajectories and Criminal Offending By Race. Am. J. Crim. Justice.

[19] Yu T., Pettit G.S., Lansford J.E., Dodge K.A., Bates J.E. (2010). The interactive effects of marital conflict and divorce on parent–adult children’s relationships. J. Marriage Fam..

[20] Yang X.Y., Anderson J.G., Yang T. (2014). Impact of Role Models and Policy Exposure on Support for Tobacco Control Policies in Hangzhou, China. Am. J. Health Behav..

[21] Parsons T. (1978). Action Theory and the Human Condition.

[22] Shilling C. (2002). Culture, the ‘sick role’and the consumption of health. Br. J. Sociol..

[23] Williams K.D. (2007). Ostracism. Annu. Rev. Psychol..

[24] Parsons T. (1975). The Sick Role and the Role of the Physician Reconsidered. Action Theory and the Human Condition.

[25] Kawachi I., Berkman L.F. (2001). Social ties and mental health. J. Urban Health.

[26] Yang X.Y., Hendley A. (2018). The gendered effects of substance use on employment stability in transitional China. Health Sociol. Rev..

[27] Yang X.Y., Kelly B., Yang T. (2020). Peer Association and Routine Activities in Sex Worker Patronage among Male Migrant Workers. Deviant Behav..

[28] Yang X.Y., Yang T. (2017). Nonmedical Prescription Drug Use Among Adults in Their Late Twenties: The Importance of Social Bonding Trajectories. J. Drug Issues.

[29] Crowley A.A. (1994). Sick child care: A developmental perspective. J. Pediatr. Health Care.

[30] Yang X.Y. (2017). Marijuana Use at Early Midlife and the Trajectories of Social Bonds. J. Dev. Life-Course Criminol..

[31] De Silva M.J., McKenzie K., Harpham T., Huttly S.R.A. (2005). Social capital and mental illness: A systematic review. J. Epidemiol. Community Health.

[32] Kawachi I., Berkman L., Kawachi I., Berkman L. (2000). Social cohesion, social capital, and health. Social Epidemiology.

[33] Zhao X., Zhang C. (2018). From isolated fence to inclusive society: The transformational disability policy in China. Disabil. Soc..

[34] Nylund-Gibson K., Choi A.Y. (2018). Ten frequently asked questions about latent class analysis. Transl. Issues Psychol. Sci..

[35] Matthews S.H. (2007). A window on the ‘new’sociology of childhood. Sociol. Compass.

[36] Moran-Ellis J. (2010). Reflections on the sociology of childhood in the UK. Curr. Sociol..

